# A Combined Raman Spectroscopic and Thermogravimetric Analysis Study on Oxidation of Coal with Different Ranks

**DOI:** 10.1155/2015/306874

**Published:** 2015-11-23

**Authors:** Weiqing Zhang, Shuguang Jiang, Christopher Hardacre, Peter Goodrich, Kai Wang, Hao Shao, Zhengyan Wu

**Affiliations:** ^1^State Key Laboratory of Coal Resources and Safe Mining, China University of Mining and Technology, Xuzhou 221116, China; ^2^School of Safety Engineering, China University of Mining and Technology, Xuzhou 221116, China; ^3^School of Chemistry and Chemical Engineering/QUILL, Queen's University Belfast, Belfast BT9 5AG, UK

## Abstract

Raman spectroscopy and nonisothermal thermogravimetric analysis (TGA) measurements have been reported for different rank coals (lignite, bituminous coal, and anthracite) and the relationship between the measurements was examined. It was found that the Raman spectra parameters can be used to characterize structure changes in the different rank coals, such as the band area ratios based on the curve-fitted results. Higher ranked coal was found to have higher values of *I*_GR_/*I*_All_ and *I*_(G + GR)_/*I*_All_ but lower values of *I*_D_/*I*_(G+GR)_, *I*_DL_/*I*_(G+GR)_, *I*_(S + SL)_/*I*_(G+GR)_, and *I*_(GL+GL')_/*I*_(G+GR)_. The oxidation properties of the coal samples were characterized by the reactivity indexes *T*_ig_, *T*_20%_, and *T*_max_ from TGA data which were found to correlate well with the band area ratios of *I*_GR_/*I*_All_, *I*_(G + GR)_/*I*_All_, and *I*_(S + SL)_/*I*_(G+GR)_. Based on these correlations, the Raman band area ratios were found to correlate with the oxidation activity of coal providing additional structural information which can be used to understand the changes in the TGA measurements.

## 1. Introduction

Coal can, and often does, undergo substantial oxidation after exposure to air under ambient conditions. This has been recognized as one of the primary reasons responsible for the self-heating of coal and, in extreme cases, the spontaneous combustion of coal in mines and stock piles [1, 2]. In general, the rate of oxygen consumption by coal decreases with an increase in the carbon content (coal rank) of the sample [3–5]. Therefore, an understanding of the relationship between the oxidation properties of coal and differences in the coal structure is important in order to determine which features are most relevant to the spontaneous combustion of coal.

Commonly, Fourier transform infrared (FTIR) spectroscopy, X-ray diffraction (XRD), and Raman spectroscopy are used in the study of the coal structure [6–13]. Raman spectroscopy provides information about both the crystalline structure and the molecular structure and thus is used most extensively [14–17]. For example, coals show a band at about 1580 cm^−1^ assigned to the stretching vibration mode with *E*_2g_ symmetry in the aromatic layers of the graphite crystallites [14, 15]. This feature, denoted by G band [6, 8], is attributed to the graphite found in higher rank anthracite type coals. For lower rank coals or disordered carbonaceous materials, an additional band appears at about 1350 cm^−1^, denoting the disordered or defect band (D band) [6, 8]. This is related to the disordered graphitic lattice vibration mode with *A*_1g_ symmetry [10, 14, 15] and represents the in-plane imperfections such as substitutional heteroatoms, grain boundaries, vacancies, or other defects in microcrystalline lattices [18, 19]. Normally, G and D bands of the highly disordered carbon materials are broad and “overlap” with each other. Thus, deconvolution of the Raman spectra is essential.

A number of detailed studies have been undertaken to assign the features observed in the Raman spectra. For example, Beyssac et al. [20] used Raman microspectroscopy with an excitation wavelength of 514.5 nm to characterize disordered and heterogeneous carbonaceous materials and assigned four bands at around 1150, 1350, 1500, and 1620 cm^−1^ to defects in poorly organized carbonaceous materials or microcrystalline graphite as well as the commonly observed G band at 1580 cm^−1^ band. Sadezky et al. [18] investigated the Raman spectra of soot and related carbonaceous materials with a Raman microscope operated at 514, 633, and 780 nm and fitted the spectra by five bands at about 1200, 1350, 1500, 1580, and 1620 cm^−1^. Sheng [16] fitted the Raman spectra of coal char, measured by a Raman microscope with an excitation wavelength of 514.5 nm, and obtained five bands around 1150, 1350, 1530, 1580, and 1620 cm^−1^ based on the results of references [18, 20]. In these reports, the bands at 1150–1200 cm^−1^ were observed in poorly crystalline carbonaceous materials and have been generally attributed to sp^2^-sp^3^ mixed sites at the periphery of crystallites or to C-C and C=C stretching vibrations of polyene-like structures [18–22]. The bands between 1500 and 1550 cm^−1^ have been assigned to amorphous sp^2^-bonded forms of carbon, such as organic molecules, fragments, or functional groups [16, 18, 20]. The 1620 cm^−1^ band was present as a shoulder on G band and is not well understood currently. However, this band was always found to be present when D band is observed and its intensity decreased with increasing degrees of order [15, 18, 20]. Sonibare et al. [23] recorded the Raman spectra of six Nigerian coals ranging from subbituminous to bituminous by a Raman microscope with an excitation wavelength of 532.21 nm and fitted the high signal intensity between G and D bands maxima with one band between 1500 and 1550 cm^−1^ and assigned this feature to amorphous sp^2^-bonded forms of carbon. Li et al. [17] measured the coal char using a Fourier transform Raman spectrometer with an excitation wavelength of 1064 nm and deconvoluted the spectra with a total of 10 bands. The 10 bands were associated with 4 main assignments, namely, G band, D band, three peaks at 1540, 1465, and 1380 cm^−1^ in the overlapping G and D bands region together assigned to 3–5 membered aromatic rings and methylene or methyl aromatic rings with mixed sp^2^-sp^3^ structures, and S band at 1185 cm^−1^ representing sp^2^-sp^3^ carbonaceous structures, which is the same as the references of [18, 20]. Whilst there is some variation in the assignments and deconvolution of the Raman features, it is clear that these measurements may be employed to determine the degree of order within the carbon structure of coal [11]. Herein, three different coal samples were examined using Raman spectroscopy to characterize the differences in the coal microstructure as a function of the coal rank. From this information, a correlation between the structural characteristics and the oxidation properties of coal was obtained.

## 2. Experimental

Three samples of coal with different rank, lignite, bituminous coal, and anthracite from China, were used as received in this study. The main coal quality parameters for each sample are summarized in Table 1. Prior to measurement, each sample was milled and sieved to a particle size of 150–250 *μ*m and then analyzed using Raman spectroscopy and thermogravimetric analysis (TGA).

The Raman spectra were measured in the range of 1000–2000 cm^−1^ using PerkinElmer RamanStation 400 F dispersive spectrometer (PerkinElmer, UK) operating at 100 mW with an excitation wavelength of 785 nm. The exposure time and number of exposures were set to 5 s and 5, respectively, operating with a sampling spot size of 100 *μ*m. To ensure that the spectra were representative of the whole of the sample, spectra from seven different samples from each rank coal were taken, and each spectrum was deconvoluted using the Omnic 8.0 package using eight Gaussian bands according to the method reported by Li et al. [17]. In the coal samples analyzed herein, a new band (~1800 cm^−1^) located on the left of GL band (~1710 cm^−1^) is more obvious compared with the spectra of coal char characterized by Li et al. [17]. Another difference is the number of curve-fitted bands between D and G bands. Typically three bands were curve-fitted in [17], while only two bands were used in this study. Curve fitting with three bands was initially examined; however, statistically nonsignificant improvement was obtained with three bands and, therefore, the minimum number of bands, that is, two, was used in the deconvolution between D and G features in the present study. For each coal, the mean value and the standard deviation of each of the band area ratios were calculated over the 7 samples of each coal measured.

TGA is one of the most commonly used methods to study the oxidation properties of coal [24, 25]. Nonisothermal TGA measurements were carried out using a thermogravimetric analyzer (TGA/DSC 1 Star System, Mettler Toledo, Switzerland) in flowing dry air at 50 cm^3^ min^−1^ at heating rates of 1 and 10°C min^−1^ over the temperature range of 25–850°C.

## 3. Results and Discussion

### 3.1. Raman Spectra

**Figure 1:**
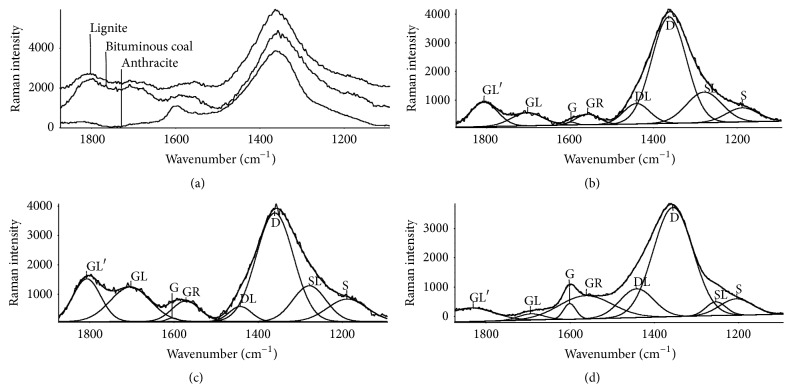
(a) Average Raman spectra of the three coals and deconvolution of the features associated with (b) lignite, (c) bituminous coal, and (d) anthracite.

Figure 1 shows average of the Raman spectra from the seven samples examined of each rank coal and the corresponding deconvolution of the features. It is shown that the Raman spectra could be successfully curve-fitted with eight Gaussian bands.

GL′ and GL bands shown in Figure 1 at 1810 and 1710 cm^−1^, respectively, represent oxygen-containing species within the structure [17]. G band at 1600 cm^−1^ has been unambiguously assigned to the crystalline graphite *E*_2g2_ vibration [13, 16–22]. GR band at 1560 cm^−1^ denotes the presence of aromatic ring systems with more than two fused benzene rings typically found in amorphous carbon structures according to [16–18, 20, 23]. It is believed that the combined intensity of G and GR bands indicates the total amount aromatic ring systems in the coal. DL band centered at 1440 cm^−1^ represents amorphous structures, such as sp^2^-bonded forms of carbon originated from organic molecules, fragments, or functional groups [16, 23]. D band at 1360 cm^−1^ shows defect structures in the graphite structure and the presence of medium-to-large sized (≥6) aromatic ring systems [13–23]. The shoulder, S, at 1190 cm^−1^ has been commonly attributed to sp^2^-sp^3^ carbonaceous structures, such as C_aromatic_-C_alkyl_, aromatic/aliphatic ethers, C-C on hydroaromatic rings, and C-H on aromatic rings [17]. SL band at 1280 cm^−1^ represents ether related structures [16–18, 20].

From Figures 1(b)–1(d), it is clear that G band is much weaker compared with D band in lignite and bituminous coal, implying poor crystallinity in these latter two coal samples. In anthracite, G band is more pronounced, showing the presence of high crystallinity. Since GR band is more intense than G band in each of the samples measured, this indicates that the fused benzene rings are the main aromatic structures within the coal. The intensities of GL′ and GL bands are more pronounced in lignite and bituminous coal than that found in anthracite, showing more oxygen-containing structures in the lower rank coals. Furthermore, S and SL bands in lignite and bituminous coals are also higher in intensity compared with anthracite indicating higher amounts of amorphous forms of carbon in the lower rank coal samples.

**Figure 2:**
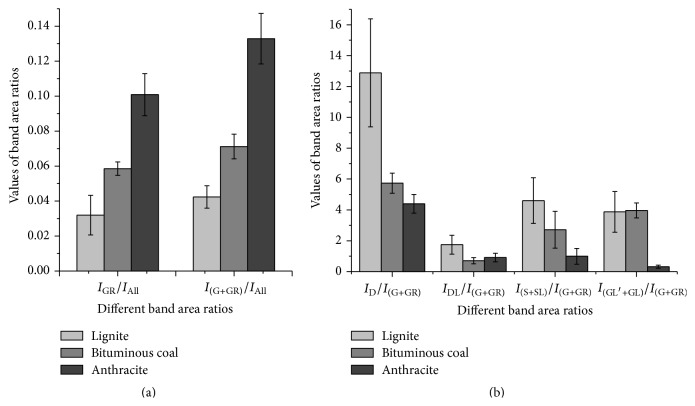
Band area ratios of (a) *I*_GR_/*I*_All_ and *I*_(G + GR)_/*I*_All_ and (b) *I*_D_/*I*_(G+GR)_, *I*_DL_/*I*_(G+GR)_, *I*_(S + SL)_/*I*_(G+GR)_, and *I*_(GL + GL′)_/*I*_(G+GR)_ for different rank coals. The error bars show the standard deviations of the parameters for each coal sample.

Further information can be obtained by analyzing the band area ratio, which is a combined parameter of the band intensity and FWHM and thus is more sensitive to the carbon structures present [16]. Therefore, the band areas associated with G and GR features were summed to provide an indication of the aromatic ring systems in the coal. Similarly, the combined band area of S and SL was obtained as this has been reported to give an indication of the defects responsible for the oxidation of the coal [16]. The combination of GL′ and GL bands represents the oxygen-containing structure in coal. The area ratios for these combinations are shown in Figure 2.

It can be seen that the higher ranked coals have higher values of *I*_GR_/*I*_All_ and *I*_(G + GR)_/*I*_All_ but lower values of *I*_D_/*I*_(G+GR)_, *I*_DL_/*I*_(G+GR)_, *I*_(S + SL)_/*I*_(G+GR)_, and *I*_(GL + GL′)_/*I*_(G+GR)_. These changes in the ratios indicate that more ordered carbon structures and structural defects/imperfections in the carbon crystallites occur as coal rank increases during coalification. This is consistent with the results reported by Nestler et al. for the structural evolution for a range of ranked coals [23, 26]. The ratio of *I*_D_/*I*_(G+GR)_, in general, shows an inverse relationship with the crystallite size of the carbon in the coal [16, 23]. The decrease in *I*_D_/*I*_(G+GR)_ with increasing coal rank indicates an increase in the average crystalline size of the coal. In addition, the decrease of *I*_(S + SL)_/*I*_(G+GR)_ with increasing coal rank demonstrates that amorphous phase of carbon transforms into a crystalline form under the coalification process. *I*_DL_/*I*_(G+GR)_ reduces significantly in both bituminous coal and anthracite compared with that found in lignite sample, showing a significant decrease in sp^2^ carbon sites of the two higher rank coals. Furthermore, the oxygen-containing structures are found to be significantly reduced in anthracite compared with lignite and bituminous coals as shown by *I*_(GL + GL′)_/*I*_(G+GR)_ value. These results also indicate a more ordered coal structure is present as the coal rank increases which leads to the increase in *I*_(G + GR)_/*I*_All_ ratio observed. These results are consistent with previously reported XRD results obtained from a range of semianthracite and bituminous coals showing the evolution of coal structure as a function of fraction of amorphous carbon, aromaticity, and crystallite size [27]. Accordingly, the band area ratios may be used for evaluating the reactivity of coals as some ratios, such as *I*_GR_/*I*_All_, *I*_(G + GR)_/*I*_All_, and *I*_(S + SL)_/*I*_(G+GR)_, change regularly with increasing coal rank.

### 3.2. TGA Results

**Figure 3:**
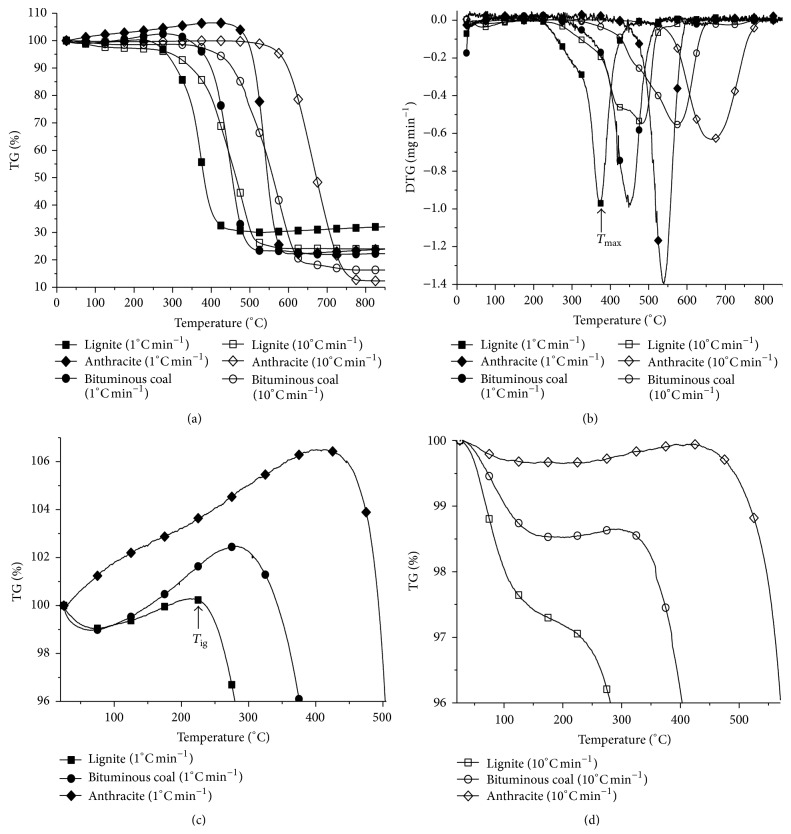
(a) TGA results of the coal samples. (b) Differential TGA (DTG) of the coal samples. (c) TGA over the range of 96–107% mass change at 1°C min^−1^. (d) TGA over the range of 96–100% mass change at 10°C min^−1^.

Figure 3 shows the TGA results of the coal samples at 1°C min^−1^ and 10°C min^−1^. As shown in Figures 3(a) and 3(b), the TGA profiles of each of the different rank coal samples are significantly different. At heating rate of 1°C min^−1^, all the coals undergo an increase in mass before a sharp mass loss which appears at elevated temperatures. This change has been reported previously and is thought to be caused by the chemisorption of oxygen on the coal surface and, thereafter, the formation of solid oxygenated surface complexes [1, 28]. From Figure 3(c), the amount of mass gained increases with increasing coal rank, which is consistent with previously reported results. Although the extent of the mass increase does give some information regarding the oxidative stability of the coal, such techniques give us limited information about the chemical nature of the coal, because the chemisorption ability of coal is closely related with the coal physical structure, which can be characterized by the porosity and internal surface area [29, 30]. In addition, these results may be complicated by volatilization of molecules from the coal which will lead to a decrease in the mass and therefore reducing the overall effect of the chemisorption of oxygen. For example, the largest mass increases anthracite which also contains the lowest amounts of volatile material (Table 1) and, therefore, it may be that the increase is more visible in this case.

For all the coal samples examined, with increasing temperature, the decomposition of the solid oxygenated complexes and direct interaction of the coal with oxygen dominates, leading to a sharp mass loss. Unsurprisingly, with increasing coal rank, the sharp mass loss shifts to higher temperatures, which shows that the oxidation stability of coal is higher as coal rank increases.  Figure 3(d) shows that, at a higher heating rate (10°C min^−1^), the mass gain was not present in lignite sample and significantly reduced in the other two coal samples compared with the profiles observed at 1°C min^−1^. This indicates that the experimental conditions, such as heating rate, can dramatically influence the TGA profiles. For example, as the heating rate increases, the corresponding coal stability moves significantly towards higher temperature and the mass gain decreases for the same rank coal (Figure 3(a)). This phenomenon is caused by the increase in thermal hysteresis in coal sample and the shorter chemical absorption time allowed at higher heating rate. However, it must be stressed that the oxidation properties of different coals must be compared under the same experimental conditions, that is, heating rate.

From the TGA and differential TGA curves, reactivity indexes which are related to the oxidation properties of coal can be calculated [31–34]. These provide a measure of the spontaneous ignition temperature of maximum coal mass (*T*_ig_), the temperature at which 20% conversion (*T*_20%_) occurs, and the peak temperature of maximum mass loss rate (*T*_max_). These results are summarized in Table 2 for each coal sample. As expected, as the rank of the coal samples increases, the reactivity index value is higher, and accordingly the coal oxidation reactivity is lower.

### 3.3. Correlation between Reactivity Indexes of Coal and Raman Band Area Ratios

**Figure 4:**
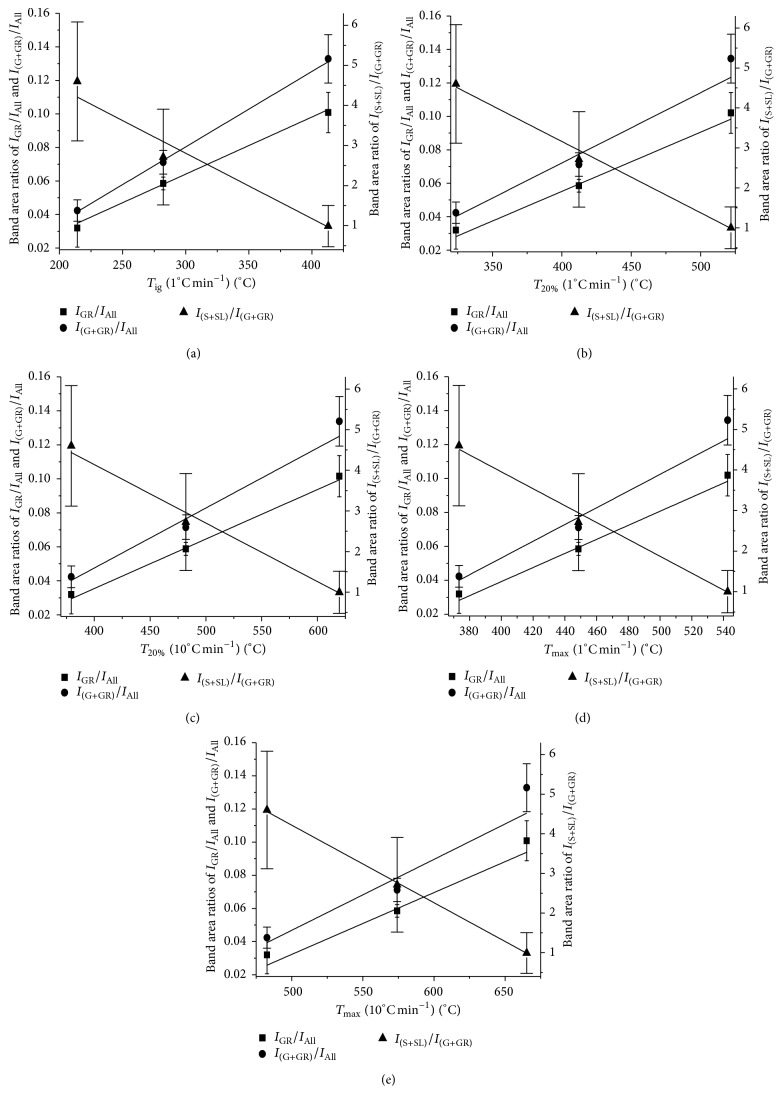
Different Raman band area ratios, *I*_GR_/*I*_All_, *I*_(G + GR)_/*I*_All_, and *I*_(S + SL)_/*I*_(G+GR)_, correlated with coal reactivity indexes: (a) *T*_ig_ (1°C min^−1^), (b) *T*_20%_ (1°C min^−1^), (c) *T*_20%_ (10°C min^−1^), (d) *T*_max_(1°C min^−1^), and (e) *T*_max_ (10°C min^−1^), respectively. The error bars show the standard deviations of the parameters for each coal sample.

In order to examine whether the Raman spectra measured and the reactivity indexes could be related, the five reactivity indexes (*T*_ig_ (1°C min^−1^), *T*_20%_ (1°C min^−1^), *T*_20%_ (10°C min^−1^), *T*_max_ (1°C min^−1^), and *T*_max_ (10°C min^−1^)) were plotted against the Raman band area ratios (*I*_GR_/*I*_All_, *I*_(G + GR)_/*I*_All_, and *I*_(S + SL)_/*I*_(G+GR)_).  Figure 4 shows that, in general, the reactivity indexes were found to increase with increases in *I*_GR_/*I*_All_ and *I*_(G + GR)_/*I*_All_ and decrease with increase in *I*_(S + SL)_/*I*_(G+GR)_. The higher integrated intensity of the defect and amorphous bands and the lower integrated intensity of G and GR bands indicate less ordering of coal crystalline structure, resulting in increased coal oxidative reactivity. Similar relationships were also found in coal char [16].

Clearer trends are found between the five reactivity indexes with *I*_GR_/*I*_All_ and *I*_(G + GR)_/*I*_All_ compared with *I*_(S + SL)_/*I*_(G+GR)_. The latter has significantly higher standard deviations which indicates a weaker relationship with the reactivity index compared with those found for *I*_GR_/*I*_All_ and *I*_(G + GR)_/*I*_All_. Therefore, this may imply that the crystalline graphite structure associated with G band and aromatic ring systems associated with GR band are more important in determining the oxidation properties of coal compared with the disordered sp^2^-sp^3^ carbonaceous structures associated with S and SL bands. Importantly, however, there is a correlation between the Raman spectra and the reactivity index for each type of coal indicating that these measurements may be used to provide a link between the structure of the coal and its oxidation.

## 4. Conclusions

Raman spectroscopy and TGA measurements were carried out for three different rank coals, lignite, bituminous coal, and anthracite, to examine whether the structural features observed could be related to the oxidation properties of the coal. Overall, the Raman spectral features showed that higher ranked coals had higher values of *I*_GR_/*I*_All_ and *I*_(G + GR)_/*I*_All_ but lower values of *I*_D_/*I*_(G+GR)_, *I*_DL_/*I*_(G+GR)_, *I*_(S + SL)_/*I*_(G+GR)_, and *I*_(GL + GL′)_/*I*_(G+GR)_, indicating an increase in the crystallinity of the coal and a decrease in the number of reacting sites and oxygen-containing structures. The changes in the intensity ratios were found to correlate with the reactivity indexes of coal obtained from TGA data. This indicated that the Raman band area ratios are related to the oxidation activity of coal and can provide additional structure information about coal.

## Figures and Tables

**Table 1:** Main coal quality indexes of coal samples.

Coal quality parameters	Samples		
	Lignite	Bituminous coal	Anthracite
Analysis (%)			
Moisture	17.75	4.44	5.06
Ash	20.86	21.59	16.55
Volatile matter	36.22	30.74	5.20
Fixed carbon	25.16	43.23	73.18
Hydrogen (%)	3.85	4.37	2.29
Heat of combustion (MJ kg^−1^)	21.18	25.47	27.36

**Table 2:** Reactivity indexes characterizing oxidation reactivity of different rank coals.

Parameter (°C)	Heating rate (°C min^−1^)	Lignite	Bituminous coal	Anthracite
*T*_ig_	1	214.17	282.20	412.93
	10	—	294.83	422.33

*T*_20%_	1	323.73	412.33	518.90
	10	379.33	481.17	617.50

*T*_max_	1	372.63	450.60	538.37
	10	481.17	573.17	665.17
